# Bacterial communities of the upper respiratory tract of turkeys

**DOI:** 10.1038/s41598-021-81984-0

**Published:** 2021-01-28

**Authors:** Olimpia Kursa, Grzegorz Tomczyk, Anna Sawicka-Durkalec, Aleksandra Giza, Magdalena Słomiany-Szwarc

**Affiliations:** 1 Department of Poultry Diseases, National Veterinary Research Institute, Al. Partyzantów 57, 24-100 Pulawy, Poland; 2 Department of Omics Analyses, National Veterinary Research Institute, Al. Partyzantów 57, 24-100 Pulawy, Poland

**Keywords:** Microbiology, Zoology

## Abstract

The respiratory tracts of turkeys play important roles in the overall health and performance of the birds. Understanding the bacterial communities present in the respiratory tracts of turkeys can be helpful to better understand the interactions between commensal or symbiotic microorganisms and other pathogenic bacteria or viral infections. The aim of this study was the characterization of the bacterial communities of upper respiratory tracks in commercial turkeys using NGS sequencing by the amplification of 16S rRNA gene with primers designed for hypervariable regions V3 and V4 (MiSeq, Illumina). From 10 phyla identified in upper respiratory tract in turkeys, the most dominated phyla were *Firmicutes* and *Proteobacteria*. Differences in composition of bacterial diversity were found at the family and genus level. At the genus level, the turkey sequences present in respiratory tract represent 144 established bacteria. Several respiratory pathogens that contribute to the development of infections in the respiratory system of birds were identified, including the presence of *Ornithobacterium* and *Mycoplasma* OTUs. These results obtained in this study supply information about bacterial composition and diversity of the turkey upper respiratory tract. Knowledge about bacteria present in the respiratory tract and the roles they can play in infections can be useful in controlling, diagnosing and treating commercial turkey flocks.

## Introduction

Next-generation sequencing has resulted in a marked increase in culture-independent studies characterizing the microbiome of humans and animals^1–6^. Much of these works have been focused on the gut microbiome of humans and other production animals^7–11^. The growing number of studies on the avian microbiome demonstrates the influence of the gastrointestinal and respiratory microbiome on the proper development and efficiency of poultry production. In recent years the studies on the bacterial microbiome of poultry have primarily focused on chickens^12–15^, particularly focused on the composition and diversity of intestinal microbiome of chickens^11,16^. Several studies have also described the gastrointestinal bacterial community in turkeys^11,17^. Less attention has been given to turkeys and their respiratory microbiome. The stability of the avian respiratory microbiome plays a critical role in preventing the colonization of pathogens. Any disruption of microbiological composition can lead to infection. Infection with bacteria such as mycoplasmas is commonly followed by a secondary bacterial or viral infection, leading to increased morbidity and mortality^18^. Infections such as coryza or infectious laryngotracheitis may be limited to the respiratory system, at least initially^19^. The pathogens could cause chronic subclinical upper respiratory infection. Bacterial species with the potential to induce infections in the respiratory tract, such as *Escherichia coli, Ornithobacterium rhinotracheale* (ORT), are often found in association with *Mycoplasma gallisepticum* or *Mycoplasma synoviae*^20,21^. In some cases, respiratory infections observed in a flock may be a component of a multisystemic disease or it may be the predominant disease with lesser involvement of other organ systems^19^.

The microbiome is a bacterial community including commensal, symbiotic and pathogenic microorganisms which usually colonize an area of host affecting its health status^4^. Understanding of the bacterial community present in the respiratory system of turkeys will allow to offer better diagnosis of many poultry diseases. Cultivation based studies helped identify pathogenic bacteria, but not all bacteria can be cultured in laboratory media. The 16S rRNA gene sequencing gives opportunities to characterize unculturable members of the Turkeys respiratory tracts. In this study we explored the bacterial communities of the upper respiratory tract (URT) of commercial turkey flocks, which will significantly broaden the knowledge about their composition and understanding of the bacterial populations in these birds.

## Results

Metagenomic methods have been used to describe the microbial community structure of URT in turkeys. We characterized bacterial composition by sequencing V3-V4 regions of 16S rRNA gene. A total of 540 swabs from nine flocks from different commercial farms were used concurrently in this study.

URT microbiome of turkey.

**Figure 1:**
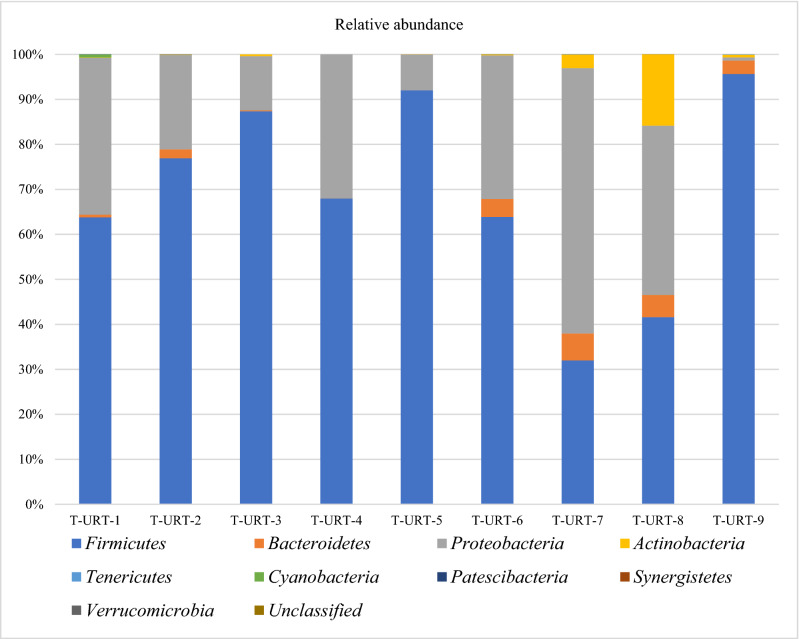
Taxonomic diversity plot showing the relative abundance of taxa at the phylum level in each sample.

**Figure 2:**
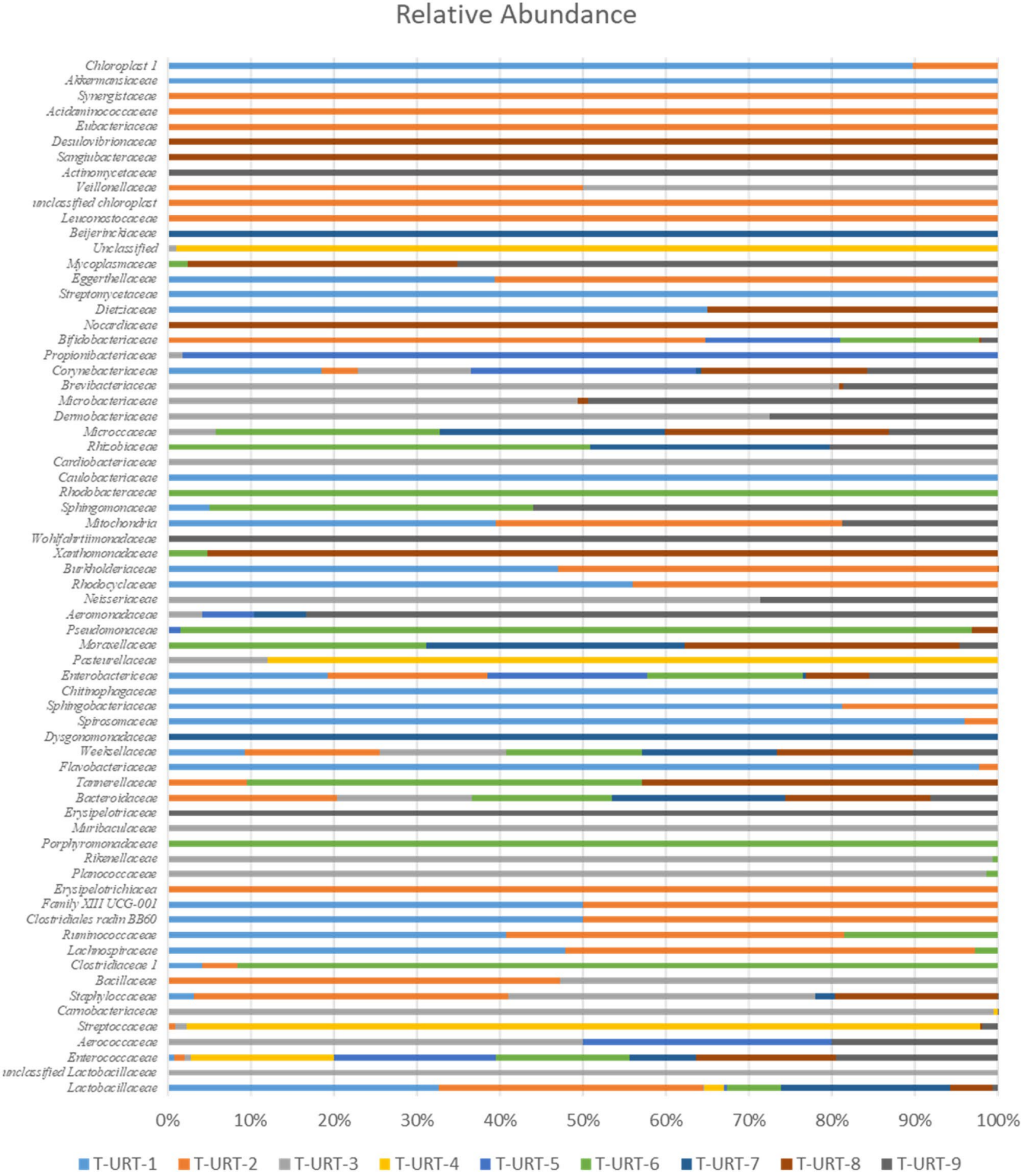
Taxonomic diversity plot showing the relative abundance of taxa at the family level in each sample.

The bacterial diversity in the URT were generally similar at the phylum level but in some cases differences in bacterial diversity were noted (Fig. 1). The sequences from turkeys represent 10 different phyla including one unclassified, 68 established bacterial family and 144 genus (Figs. 1, 2). Significant differences in microbial composition along the turkeys URT at the class (Kruskal–Wallis test, *p* = 0.042) and family (Kruskal–Wallis test, *p* = 0.0067) level were observed (Fig. 2, Additional file 1). Over 99% of the URT microbiota of the turkey flocks were comprised of *Firmicutes* (69.11% ± 20.53%), *Proteobacteria* (26.41% ± 16.90%), *Bacteroidetes* (2.31% ± 2.17%), *Actinobacteria* (2.26% ± 4.94%), *Tenericutes* (0.015% ± 0.03%), *Cyanobacteria* (0.087% ± 0.218%) and unclassified phylum (0.002% ± 0.004%). In one flock (T-URT-1) a very small number of bacteria belonging to phylum *Patescibacteria* (0.003%) was found. However, in another flock—T-URT-2 was also a small number of bacteria from two different phyla: *Synergistetes* (0.01%) and *Verrucomicrobia* (0.01%). The microbiomes of T-URT-7, T-URT-8 and T-URT-9 have a higher abundance of operational taxonomic units (OTUs) from *Bacteroidetes* and *Actinobacteria* phylum than others flocks. In addition, the microbiome of T-URT-9 was dominated by bacteria from the *Firmicutes* phylum (Fig. 1). The most common bacterial OTUs in this flock were *Enterococcus* but also classified OTUs *Actinobacter, Psychrobacter, Neisseria* and also into the species level: ORT and *M. gallisepticum.*

**Figure 3:**
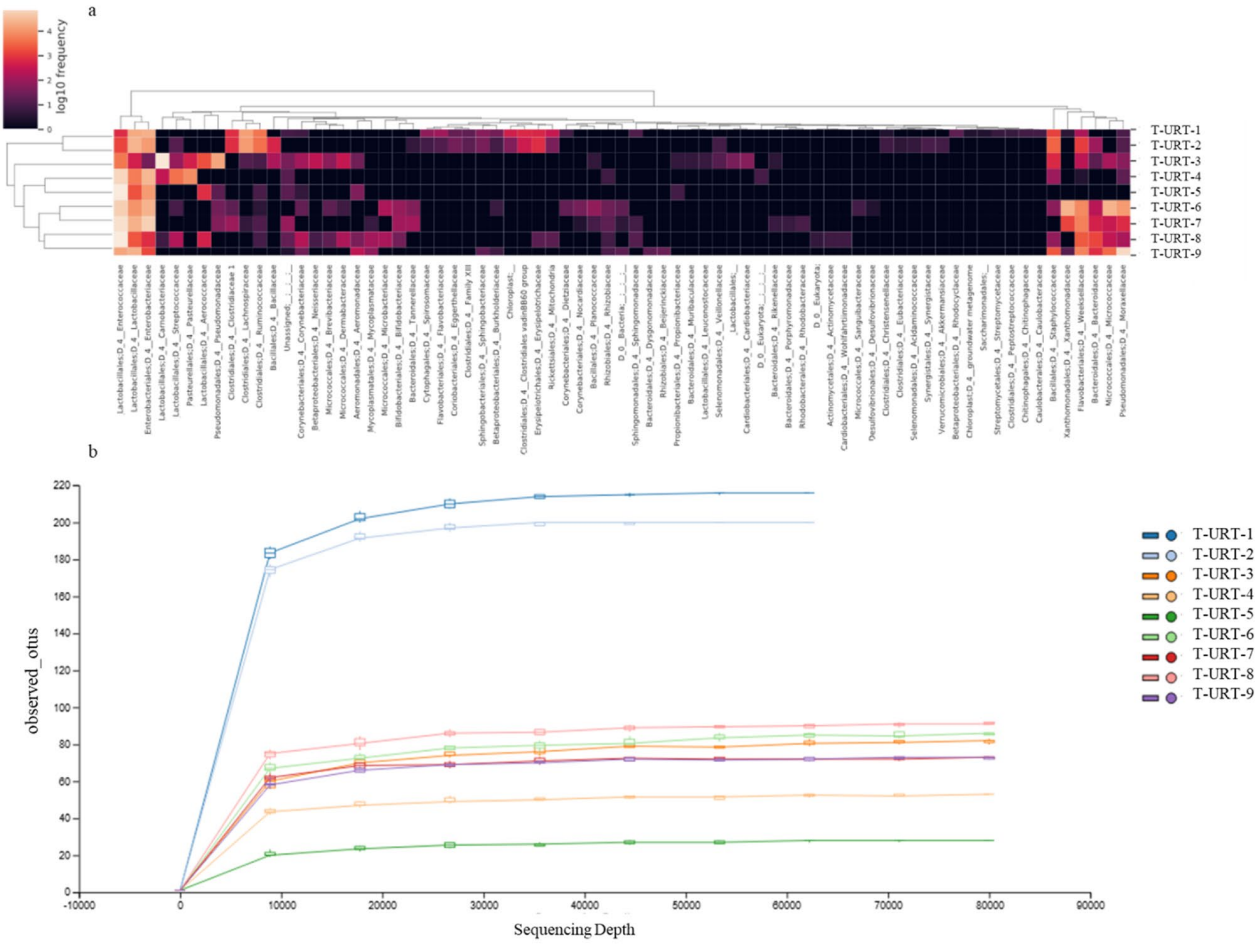
Heatmap of bacterial OTUs in turkeys URT. (**a**) Heatmap of bacterial OTUs in the upper respiratory tract of turkeys. Heatmap depicting abundance of all 73 OTUs by overall abundance across samples. Normalized heatmap on taxonomic level 5 was constructed with clustering on both samples and feature axes. (**b**) Observed OTUs comparing URT flocks.

In all flocks *Firmicutes* was dominated by *Bacilli* and *Clostridia, Proteobacteria* was largely represented by *Gammaproteobacteria* and *Alphaproteobacteria. Bacteroidetes* consistent only of the class *Bacteroidia. Actinobacteria* was the most abundant classes in the phylum *Actinobacteria.* By comparing the microbiome datasets between the flocks we identified several bacterial classes, including *Bacilli* (62.33 ± 23.84%) and *Clostridia* (6.57 ± 12.27%), that were differentially expressed (Additional file 1). On average, the most common bacterial OTUs found in URT samples were *Enterococcus* (38.78 ± 35.1%), *Lactobacillus* (12.48 ± 13.21%), *Escherichia-Shigella* (12.04 ± 11.72%), *Pseudomonas* (1.27 ± 3.44%) and unclassified *Enterobacteriaceae* (0.93 ± 2.5%) (Fig. 3 and Additional files 2 and 3 online). *Enterococcus* were found more prominently in older turkeys, while *Lactobacillus* were found more in younger turkeys (Fig. 3a, Table 2). The number of observed OTUs were higher in flocks T-URT-1 and T-URT-2 from the Lubelskie province compared to the rest of the flocks. The lowest number of observed OTUs were in the T-URT-5 flock from the Warmińsko—Wazurskie province (Fig. 3b).

Upon examining the bacterial composition of Turkeys flocks, we found that in each sample are bacteria belonging to a small number of taxonomic classifications. The tracheal swabs included OTUs classified as *Ornithobacterium* (1.58 ± 1.87%)*, Mycoplasma* (0.01 ± 0.02%)*, Gallibacterium* (0.81 ± 2.19%)*, Avibacterium* (0.01 ± 0.03%), *Staphylococcus* (1.05 ± 1.6%) and *Streptococcus* (0.36 ± 0.93%) (Table 1 and Additional files 2 and 3 online). The majority of the reads were not distinguishable to the genus node.

**Table 1:** Relative abundance of selected OTUs at the genus level in turkeys URT.

ID	*Mycoplasma*	*Ornithobacterium*	*Gallibacterium*	*Avibacterium*	*Staphylococcus*	*Streptococcus*
T-URT-1	–	–	–	–	2%	–
T-URT-2	–	–	–	–	5%	0.05%
T-URT-3	–	0.2%	7%	–	0.09%	3%
T-URT-4	–	–	0.3%	0.1%	0.3%	0.07%
T-URT-5	–	–	–	–	–	–
T-URT-6	0.003%	3%	–	–	–	–
T-URT-7	0.05%	5%	–	–	2%	–
T-URT-8	–	4%	–	–	0.03%	0.01%
T-URT-9	0.08%	2%	–	–	0.05%	0.1%

In order to evaluate bacterial differences between samples, we analyzed the β -diversity based on unweighted UniFrac. We analyzed the bacterial compositions of the samples for the presence of two pathogens ORT and *Mycoplasmas* that may affect the respiratory system and the general health of birds. The difference in bacterial community were visualized by principal coordinate analysis graph (PCoA) based on the weighted UniFrac distance matrix (Additional file 4c, d). PCoA graph showing that the age of flocks with *Mycoplasma* OTUs were at 3 to 22 weeks (T-URT-6, T-URT-7, T-URT-9) and birds with ORT OTUs were at 3–30 weeks (T-URT-3, T-URT-6, T-URT-7, T-URT-8, T-URT-9) (Additional file 4c,d, Table 2).

**Table 2:** Flock metadata.

ID of sample	Location	Age (week)	Year of sampling	Type of samples
T-URT-1	Lubelskie	3	2020	scientific
T-URT-2	Lubelskie	3	2020	scientific
T-URT-3	Warmińsko-Mazurskie	30	2019	diagnostic
T-URT-4	Warmińsko-Mazurskie	52	2017	monitoring
T-URT-5	Warmińsko-Mazurskie	3	2017	diagnostic
T-URT-6	Wielkopolskie	22	2017	diagnostic
T-URT-7	Warmińsko-Mazurskie	3	2019	monitoring
T-URT-8	Kujawsko-Pomorskie	8	2017	diagnostic
T-URT-9	Kujawsko-Pomorskie	6	2019	diagnostic

**Figure 4:**
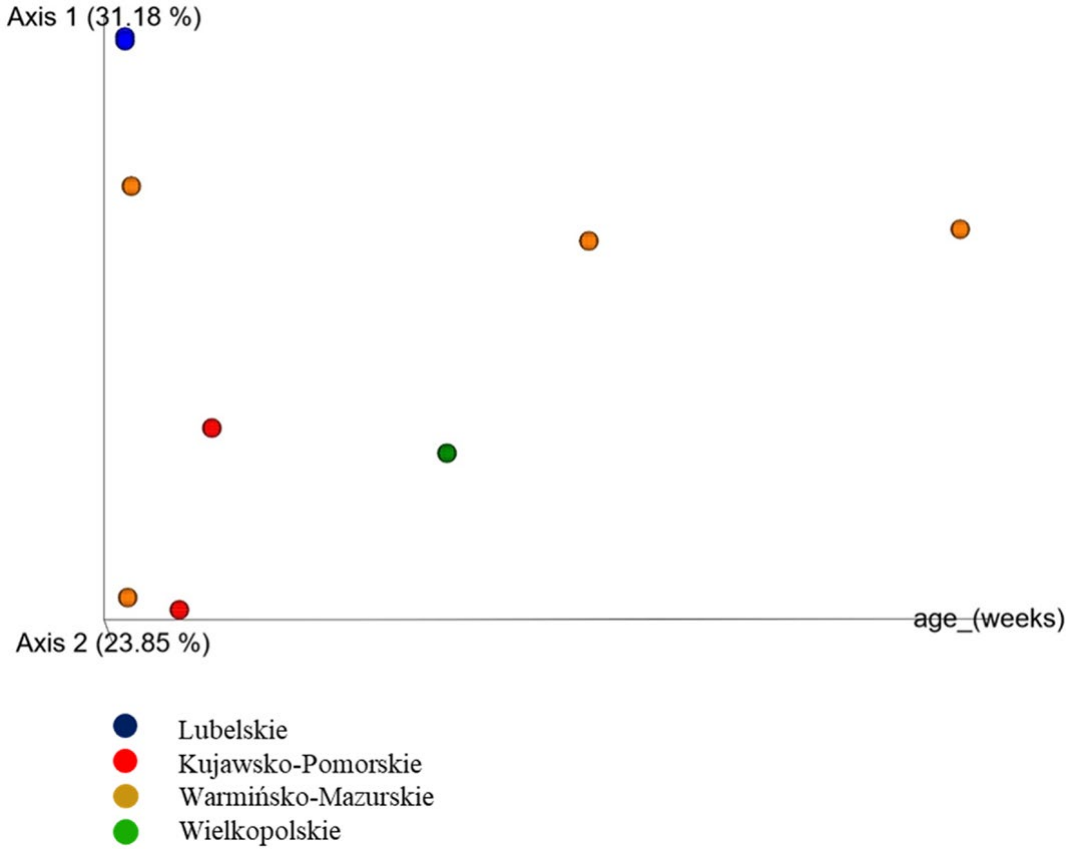
PCoA graph showing clustering by bacterial community composition in tracheal swabs from turkeys of different ages according geographical localization.

A Venn diagram was constructed to reveal bacterial OTUs at the genera level that were unique or shared between different flocks (Additional file 4a,b). Among of the 144 phylotypes only 2 OTUs were observed in *Mycoplasma* positive and negative turkey flocks. Only 4 OTUs were shared between flocks *Mycoplasmas* positive and ORT positive. The samples used in this study were geographically diverse and collected from flocks of different age. The richness of microbial communities were various in tracheal swabs. The tracheal community composition shifted very gradually as the turkeys aged. The bacterial diversity was also different between birds in the same age. PCoA graph show that only samples from the Lubelskie province (blue color) are very close to each other. The samples were taken at the same time and from flocks of the same age and their bacterial diversity seem to be similar (Fig. 4).

**Figure 5:**
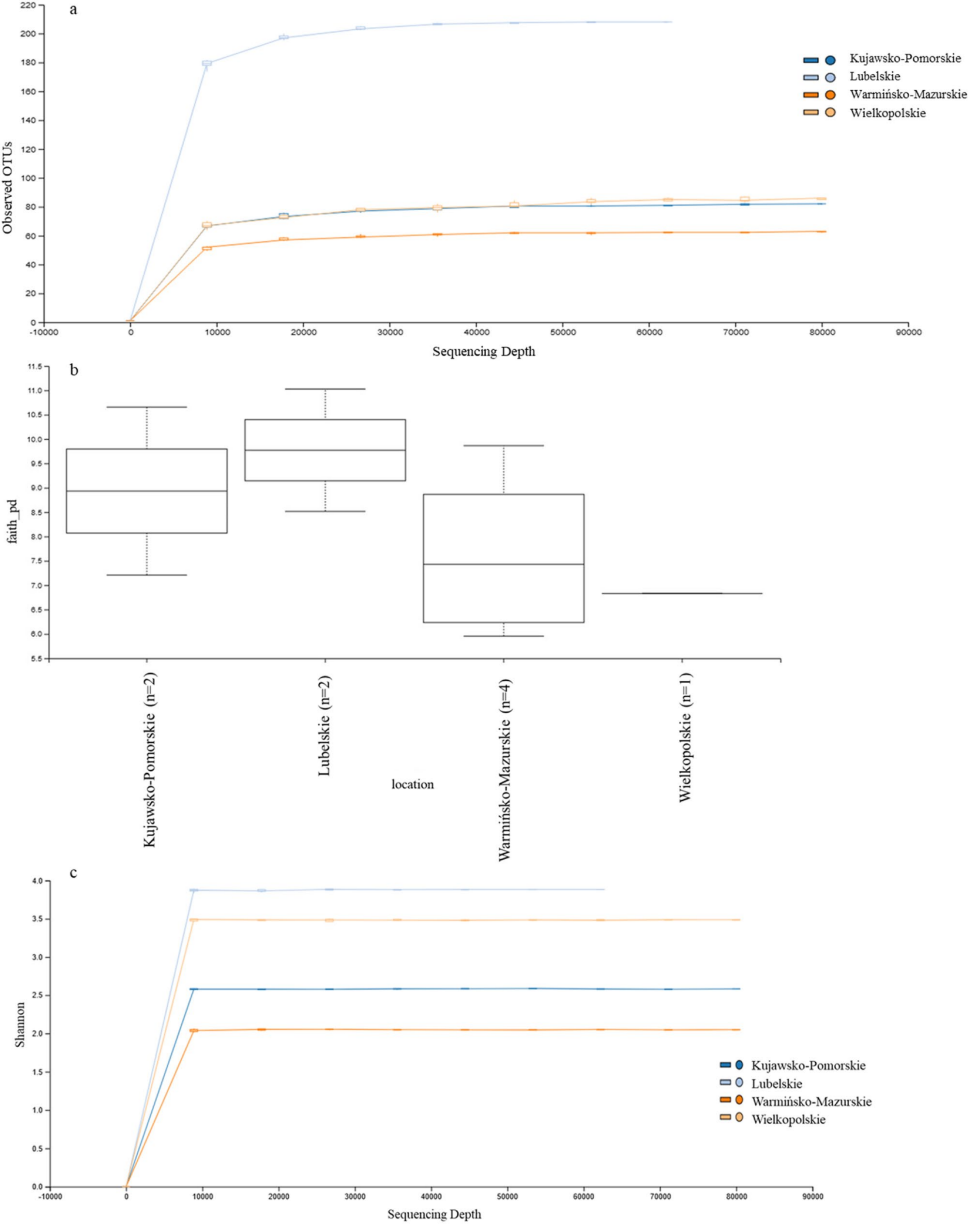
Comparison of URT microbiome of turkeys. (**a**) Observed OTUs comparing geographical location. (**b**) The Faith PD boxplots. (**c**) Shannon diversity index comparing samples from different geographical location.

**Figure 6:**
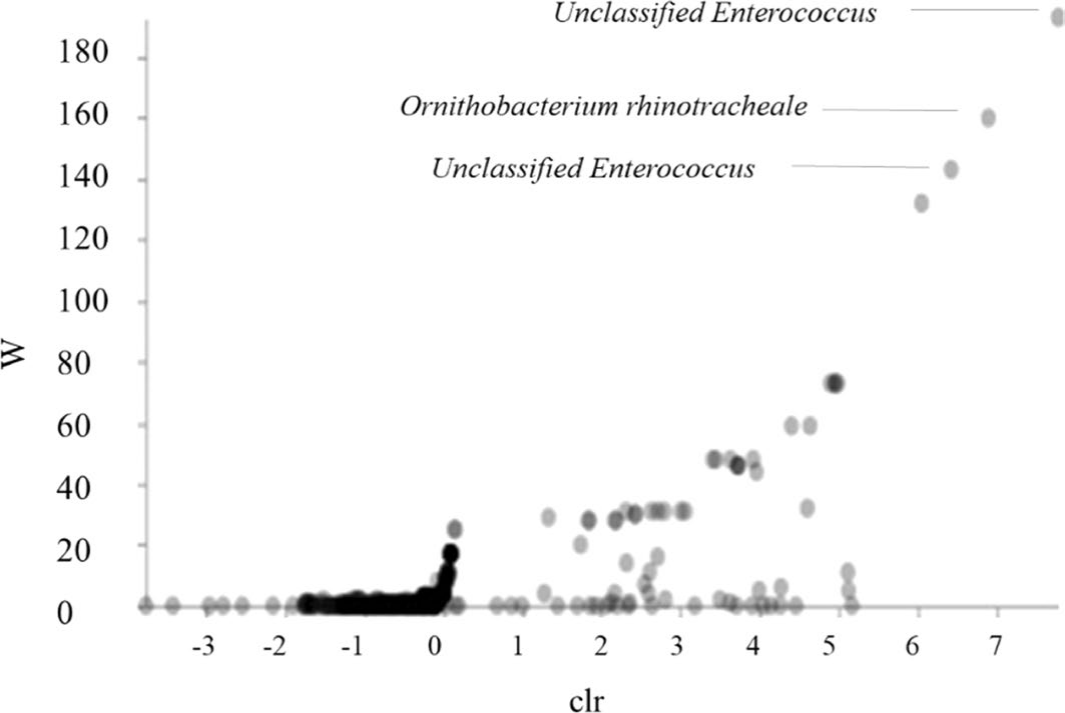
ANCOM Volcano Plot. In the ANCOM analysis the W value represents the number of times of the null-hypothesis (the average abundance of a given species in a group is equal to that in the other group), it was rejected for a given species. The clr (axe x) is the central log ratio.

In Faith phylogenetic diversity (PD), based on the geographical localization there was no significant difference between flocks (Kruskal–Wallis Test; *p* > 0.05). Samples were collected across four different provinces in Poland (Kujawsko-Pomorskie, Warmińsko-Mazurskie, Wielkopolskie i Lubelskie). The Faith PD index value of the group of flocks from Lubelskie province were higher than those from three others province (Fig. 5a). Samples from Warmińsko-Mazurskie province showed large individual variance (Fig. 5b). The Shannon index value of the samples from the Lubelskie and Wielkopolskie provinces were higher than value of the samples from the Kujawsko-Pomorskie and Warmińsko-Mazurskie provinces, respectively (Fig. 5c). We performed a composition analysis of microbiome (ANCOM) to identify key genera discriminating the microbiota of turkey flocks. From all of the observed OTUs at the genus level, two unclassified *Enterococcus* (W = 193), (W = 143) and ORT (W = 160) showed a significant difference (*p* < 0.05) in abundance between the microbiome in the URT in *Mycoplasma* positive flocks (Fig. 6).

## Discussion

Understanding the poultry microbiome has the potential to offer better diagnosis and rational management of many poultry diseases including the use of antibiotics. New sequencing technologies enable us to characterize the turkey’s respiratory microbial communities without using traditional culturing techniques. The analysis of the variable regions of the 16S rRNA gene of whole bacterial community from tracheal swabs samples gives the possibility of detecting pathogens without the need for culture. Some of the respiratory pathogens are carried in the healthy flocks and the factors that cause the switch between carriage and disease (such as inhibition by commensal bacteria or intercurrent viral infections) are only partly understood. Most of the current research on the commensal bacteria of poultry has been mainly focused on the gut microbiome of chicken, and less attention has been given to turkeys and respiratory microbiome.

In this study, we tested samples from turkey flocks of different ages and geographically distinct farms in Poland. We identified bacterial profiles of URT of turkey and provided new insights for further identification of novel pathogens in farm flocks.

Based on the results of this study, a number of avian commensals as well as pathogens belonging predominantly to the phyla *Firmicutes, Proteobacteria, Bacteroidetes, Actinobacteria,* and *Tenericutes* have been reported (Fig. 1). These are the main phyla in the respiratory tract reported also in domestic and wild birds^22,23^, and similarly like those present in the respiratory tract microbiome of other animals^24,25^. At the genus level *Lactobacillus, Enterococcus, Escherichia-Shigella,* and *Morganella* were most abundant and represented the bacterial genus in the respiratory tract of turkeys. In this study, 68 taxa at the family level were observed (Figs. 2, 3). One hundred and forty-four genera were found in the URT of turkeys. Results of the study on the digestive system of chickens and turkeys demonstrated that age and environment appear to play a key role in the initial stages of the turkey bacterial microbiome maturation^17,26^. Just as the bacterial composition of the respiratory tract in chickens varies with age, and the same may be in the turkeys^12,15^. As in chickens, older turkeys may be exposed to a number of management stressors and physiological changes as the birds mature and enter the laying period which can affect their microbiome. In other studies, significant differences in the richness and diversity of respiratory track communities were observed between age groups, but in the nasal cavity of chickens^15^. However, the tracheal community composition shifted very gradually as the chicken aged as in our study with turkeys. In this study, tracheal swabs samples were analyzed at the OTU level for specific subsets of OTUs representing geographical localization of sampling and age of birds (Figs. 4, 5). The richness of the microbial communities in some flocks was decreasing with age, but flocks did not differ significantly in diversity in the phylum level (AMOVA). Analysis clearly showed that there were small number of shared subsets of OTUs present across all samples (Additional file 4a,b). We observed differences in the bacterial communities in the class and family OTUs based upon the age of the turkey from which the samples were taken. Interestingly, the samples did not cluster by age group according to their bacterial diversity (Fig. 4). Only samples from the Lubelskie province collected at the same time and from flocks of the same age show similar bacterial diversity. Fluctuations in the differentiation of the microbiome in older birds may be due to the continued exposure to numerous management stressors and physiological changes during maturation of the birds. Factors related to the new environment, change of nutrition or treatment with antibiotics may also affect differences in the bacterial composition of the respiratory system of turkeys. Additionally, there is no data available on other turkey upper respiratory tract studies that could be used to compare composition and variability of the bacterial communities across age groups. Nevertheless, in order to confirm these results and extend of the URT analysis, in future work an investigation on a large number of samples will be needed.

In turkeys, as in chickens, the most common bacteria identified in the respiratory tract were members of the *Lactobacillales* and along with members of the *Enterobacteriales*^12,27^. In this study, *Lactobacillus* and *Enterococcus* were detected in all URT samples; although their relative abundance was varied in some flocks. Some OTUs displayed a temporal trend and were found more prominently in older turkeys, such as those classified as *Enterococcus,* while *Lactobacillus* were found more in the youngest turkeys (Fig. 3a).

The microbial analysis suggests that the URT bacterial communities are different from both the intestinal and litter samples. However, the identification of isolated bacteria indicates that both environments are partially reflected in URT. The data from this study indicates that the turkey URT bacterial communities through contact with the litter environment, are exposed to colonization with microorganisms showing a preference for the respiratory system. Bacteria that were already been identified as members of the turkeys gut microbiota, such as *Ruminococcus, Brachybacterium, Virgibacillus, Blautia*, *Weissella, Brevibacterium, Staphylococcus, Clostridium* and *Corynebacterium* were also isolated in this study^11,17^. These bacteria were found in two sections of the gastrointestinal tract in the ileum and litter samples but were not present in cecum samples. Bacteria previously isolated from poultry house air, such as *Jeotgalicoccus*^28^ were also found in the respiratory tract in chickens^12^ and now also in URT of turkeys (Additional file 2 and 3 online).

We note that flocks T-URT-1 and T-URT-2 contained a more diverse microbiome than the other flocks (Figs. 2, 3 and Additional file 1). The cause of this could be various factors such as housing and environmental conditions, age, or performance stress. In the URT of these flocks a small abundance of bacteria from three phyla: *Patescibacteria, Verrucomicrobia* and *Synergistetes* were found*.* But, to the best of our knowledge, representatives of these phyla have not been previously attributed to the respiratory system of turkeys. In previous publications the phyla *Verrucomicrobia* and *Synergistetes* were identified in the gut of chickens^11^. The bacteria from the family *Akkermansiaceae* (phylum *Verrucomicrobia*) are mucin-degrading bacteria and may be the most prestigious microorganisms among the next-generation probiotics^24^. The bacteria from phylum *Synergistetes* are mostly anaerobic microorganisms. These bacteria can be found both in the animal digestive tracts and in sites of human diseases occur (areas of periodontal disease). They were also found in the soil and in wastewater treatment plants disease^29^. The *Patescibacteria* phylum is the newly defined superphylum and has been found to be prevalent in lake, sediment, groundwater different aquifer environments and also in soybean. This superphylum has small genomes and a presumed symbiotic or parasitic lifestyle^30,31^. However, we also do not know the role of bacteria that we found as unclassified at the phylum level, therefore a further study of respiratory microbial communities of turkeys is needed.

Several bacterial taxa identified in this study have been described as positively affecting chicken flock performance. These include *Bacteroides, Faecalibacterium, Parabacteroides* in the gut; and *Bifidobacterium, Corynebacterium, Dietzia, Staphylococcus* in the trachea^25^. In another study it was found that the rate at which the chickens process their feed (feed conversion rate) is correlated with the presence of *Faecalibacterium*. Other studies describe *Bacteroides* as a major component of the poultry microbiome and point to their possible probiotic capabilities^11,25,32,33^.

In the URT of turkeys, potential respiratory pathogens including *Avibacterium, Gallibacterium, Mycoplasma*, and *Ornithobacterium* were found (Table 1). Multifactorial respiratory disease in poultry is often associated with bacterial factors including *E. coli*, ORT, *M. gallisepticum, M. synoviae,* which are frequently implicated. The most pathogenic bacterial species causing significant diseases of poultry are *Mycoplasma gallisepticum* (mycoplasmosis) and ORT (respiratory disease)^22^. Comparing the bacterial composition of the URT of turkey flocks with and without *Mycoplasma* and *Ornithobacterium*, four shared genera were found. Two genera were shared in the URT of turkeys between flocks with and without *Mycoplasma*. The presence of these pathogens may lead to occurrence clinical symptoms and deterioration of the health of the birds. The upper respiratory tract is a reservoir of opportunistic pathogens, which can proliferate and infect the air sacs when the poultry immune system is compromised due to stress or primary viral infections^24^. Interestingly, most birds at the time of sampling did not display respiratory signs of clinical disease. One flock T-URT-9 had neurological symptoms and weak respiratory signs. Such neurological problems have been reported in turkeys due to meningoencephalitis caused by MG neurotropic strains^34^. The presence of ORT can also induce osteomyelitis of the cranial bones in turkeys causing nervous signs, movement disturbances and recumbence^35^. Community level similarities in the URT of samples were compared using principal coordinates analysis (Additional file 4c, d). Samples were stratified primarily by the age of flock. Upon visualization of the PCoA plots, *Mycoplasma* positive and ORT positive samples did not overlap and were distinct from each other. However, all sample types shifted similarly over time on the plot, indicating that bird age has an impact on the bacterial communities (Additional file 4). This highlights the potential importance of these pathogens in the environment, causing also subclinical disease and subsequently impacting the performance of birds. Therefore, the interaction between turkeys and these species should be studied to understand the function of the respiratory tract of turkeys.

Our preliminary study identified the bacterial profiles of turkey respiratory tracts and provided new insights for further identification of novel pathogens in flocks of these birds species. The further studies on a larger sample size are necessary to ascertain the validity of these findings. To the best of our knowledge, this is the first study to examine the bacterial communities of the URT of turkeys. We determined that in the URT of turkeys were OTUs of different bacterial species. Part of the these were not previously associated with the respiratory tracts of birds. The identified phyla were present in relatively low proportions. In this study we also identify many novel organisms which are yet unclassified. Bacteria unknown or previously considered non-pathogenic can cause health problems in flocks of turkeys. This bacteria may play an important role in the development of infection.

The avian respiratory tract is the common site of viral pathogen entry and disease, including Newcastle disease, infectious bronchitis or avian influenza which are dangerous to the health and life of birds. Therefore, it is very important for the poultry industry to prevent poultry respiratory infections.

## Methods

### Sample collection

Samples were collected from commercial turkeys from geographically distinct farms located in Poland. Respiratory samples consisted of trachea swabs (Medlab Products, Poland) that were collected from 60 birds per flock. Tracheal swabs were taken with sterile swabs to avoid possibility of transferring environmental bacteria. Most birds at the time of sampling did not display lesions or other respiratory signs of clinical disease, only one flock (T-URT-9) had neurological symptoms and weak respiratory signs. Swab samples were shipped to the National Veterinary Research Institute as part of a monitoring program or diagnostic tests. Relevant flocks metadata including age, year of sampling and location of flocks are in Table 2. All samples were suspended in sterile phosphate buffered saline (PBS) (1 ml PBS per one swab and pooled) and stored at − 20 °C. Part of the suspension was centrifuged for 10 min at 10,000 rpm. The supernatant was carefully removed and the pellets were suspended in 800 μl PBS. The supernatant was used for DNA isolation.

The samples were collected from animals by authorized veterinarians during clinical studies following standard procedures. All methods used in this study were carried out in accordance with relevant guidelines and regulations. The animals are not directly involved in this study. According to the Local Ethical Committee on Animal Testing and Directive 2010/63/EU on the protection of animals used for scientific purposes (Chapter I, article 1, p. 5 b, d, f) the formal ethical approval is not required for this kind of study.

### DNA extraction and16S rRNA gene sequencing

Genomic DNA was isolated from each pooled sample with the use of Maxwell RSC PureFood Pathogen Kit (Promega, USA) according to the manufacturer’s recommendations. The quantity and quality of the DNA was determined using the Nanodrop 1000 system (Thermo Scientific). DNA extraction from the PBS used for sample preparation was conducted as a negative control. Briefly, before starting extraction, 50 µl lysozyme (10 mg/ml, Novazym), 6 µl mutanolysin (5KU/ml, Sigma-Aldrich), and 8 µl lysostaphin (5 g/ml, Sigma-Aldrich) were added to the samples followed by incubation for 45 min at 37 °C. All 16S libraries were prepared using Illumina metataxonomic protocol: “16S Metagenomic Sequencing Library Preparation”^36^. The V3-V4 regions of 16S rRNA gene were amplified using 2 × KAPA HiFi Hot Start Ready Mix (Roche) and primers:

16S Amplicon PCR Forward Primer:

#### 5′TCGTCGGCAGCGTCAGATGTGTATAAGAGACAGCCTACGGGNGGCWGCAG

16S Amplicon PCR Reverse Primer:

#### 5′GTCTCGTGGGCTCGGAGATGTGTATAAGAGACAGGACTACHVGGGTATCTAATCC

The length of targeted region is approximately 460 bp. Primers include overhang adapter sequences, which are compatible with Illumina index and sequencing adapters. PCR was conducted according to the manufacturer’s recommendations. Products were checked on Fragment Analyzer using kit: dsDNA 935 Reagent Kit. Clean up step was performed using AMPure XP beads (Beckman Coulter), according to the protocol. Index PCR step was carried out with use of 2 × KAPA HiFi Hot Start ReadyMix (Roche) and dual Index adapters (Illumina), according to the manufacturer’s recommendations. Clean up step was again performed using AMPure XP beads (Beckman Coulter), according to the protocol. Libraries were checked and average libraries sizes were determined on Fragment Analyzer, using kit: dsDNA 935 Reagent Kit. Quantification of the libraries was carried out with use of Qubit 3.0 fluorometer (Thermo Fisher Scientific). Normalization of the libraries was performed according to the protocol, libraries were pooled in equimolar concentration and then denatured according to the Illumina protocol and diluted to the final concentration of 20 pmol. The diversity of the run was ensured by compositing metataxonomic with WGS Illumina libraries. The sequencing run was performed on Illumina MiSeq platform and MiSeq reagent kit V3 (600 cycles) with paired reads.

### 16S rRNA gene taxonomy assignments

The 16S rRNA gene sequencing data was processed through the Quantitative Insights into Microbial Ecology (QIIME 2Core 2020.2)^37^ and Krona^38^. The sequences were clustered into operational taxonomic units (OTUs) using dada2 denoise-paired method with parameters: –p-trim-left m which trims off the first m bases of each sequence (Illumina indexes trimming); –p-trunc-len n which truncates each sequence at position n (forward reads n = 300, reverse reads n = 242) allowing to remove regions of sequences below quality score 15. During this step, reads were also corrected and chimeric sequences filtered. Trained Silva 132 99% OTUs (full-length) classifier was used to assign taxonomy to sequences. Sampling depth was even to 62,338 to ensure that every sample was taken into consideration in analysis. Of the 11 sample sequencing data set, two flock showing different composition compared to other flock, were excluded from analysis.

To compare and illustrate the overall URT microbial community structures of the turkeys flocks, Krona charts were generated, that allow comparison between microbiomes based on detailed phylogenetic composition. Krona charts were generated using the—krona_qiime.py from Qiime2_pipeline_IT_EMP.md (https://github.com/lokeshbio/AmpliSeq/blob/master/Qiime2_pipeline_IT_EMP.md#krona-plots). The Venn diagrams, including all OTUs generated by the OTU picking step, were calculated using the website Bioinformatics & Evolutionary Genomics (http://bioinformatics.psb.ugent.be/webtools/Venn).The metadata of flocks and corresponding taxonomic classifications in Krona charts have been included as Additional files 2 and 3 respectively.

### Statistical analysis of the URT microbiome of Turkeys

Alpha diversity was measured using the Shannon index. Beta-diversity was determined using unweighted based only on the presence/absence of taxa and measures the distance between two communities, and weighted UniFrac analysis that is a quantitative measurement accounting for differences in the relative abundance of OTUs between different communities. Both analyzes are very sensitive and detect the smallest differences in the microbiome^39^. The relative taxa abundance of the flocks is presented as a mean % value. To identify differentially abundant taxa and assess the association between the microbial community of the upper respiratory tract of turkeys, ANCOM analysis implemented in QIIME2 was performed. The Kruskal–Wallis test was used to detect significant differences in richness and diversity between bacterial communities present in URT in turkeys. Construction of heatmaps was performed using the QIIME2 feature-table plugin. PCoA graphs were constructed to visualize sample clustering by bacterial community composition. A Venn diagram was constructed to reveal bacterial OTUs at the genus level that were unique or shared between different flocks. The OTUs observed in any samples in a flock were counted.

## Supplementary Information

Supplementary Information 1.

Supplementary Information 2.

Supplementary Information 3.

Supplementary Information 4.

## Data Availability

The sequences from the metagenomic libraries were deposited in the NCBI Sequence Read Archive under BioProject accession number: PRJNA644253.
