# Supplementary material for: Bacterial communities of the upper respiratory tract of turkeys
Source: Sci Rep. 2021 Jan 28;11:2544. doi: 10.1038/s41598-021-81984-0 (PMC7843632; doi:10.1038/s41598-021-81984-0)
Supplement: Supplementary file 1 — Supplementary Information 1. [file 41598_2021_81984_MOESM1_ESM.docx]

**Bacterial communities of the upper respiratory tract of turkeys**

Olimpia Kursa^1,*^, Grzegorz Tomczyk^1^, Anna Sawicka-Durkalec^1^, Aleksandra Giza^2^, Magdalena Słomiany-Szwarc^2^

1. Additional file 1. Taxonomic diversity plot showing the relative abundance of taxa at the class level in each sample
