# Supplementary material for: Bacterial communities of the upper respiratory tract of turkeys
Source: Sci Rep. 2021 Jan 28;11:2544. doi: 10.1038/s41598-021-81984-0 (PMC7843632; doi:10.1038/s41598-021-81984-0)
Supplement: Supplementary file 2 — Supplementary Information 2. [file 41598_2021_81984_MOESM2_ESM.docx]

**Bacterial communities of the upper respiratory tract of turkeys**

Olimpia Kursa^1,*^, Grzegorz Tomczyk^1^, Anna Sawicka-Durkalec^1^, Aleksandra Giza^2^, Magdalena Słomiany-Szwarc^2^

Additional file 2 – KRONA charts - file online

Additional file 3 – ID of flocks in Krona file

| **ID flock** | **KRONA ID run** |
| --- | --- |
| T-URT-1 | 2275 |
| T-URT-2 | 2276 |
| T-URT-3 | 2382 |
| T-URT-4 | 2381 |
| T-URT-5 | 2388 |
| T-URT-6 | 2384 |
| T-URT-7 | 2387 |
| T-URT-8 | 2383 |
| T-URT-9 | 2345 |
