# Supplementary material for: Bacterial communities of the upper respiratory tract of turkeys
Source: Sci Rep. 2021 Jan 28;11:2544. doi: 10.1038/s41598-021-81984-0 (PMC7843632; doi:10.1038/s41598-021-81984-0)
Supplement: Supplementary file 3 — Supplementary Information 3. [file 41598_2021_81984_MOESM3_ESM.docx]

**Bacterial communities of the upper respiratory tract of turkeys**

Olimpia Kursa^1,*^, Grzegorz Tomczyk^1^, Anna Sawicka-Durkalec^1^, Aleksandra Giza^2^, Magdalena Słomiany-Szwarc^2^

Additional file 4. Shared and unique OTUs at the genus level in the URT of turkeys.


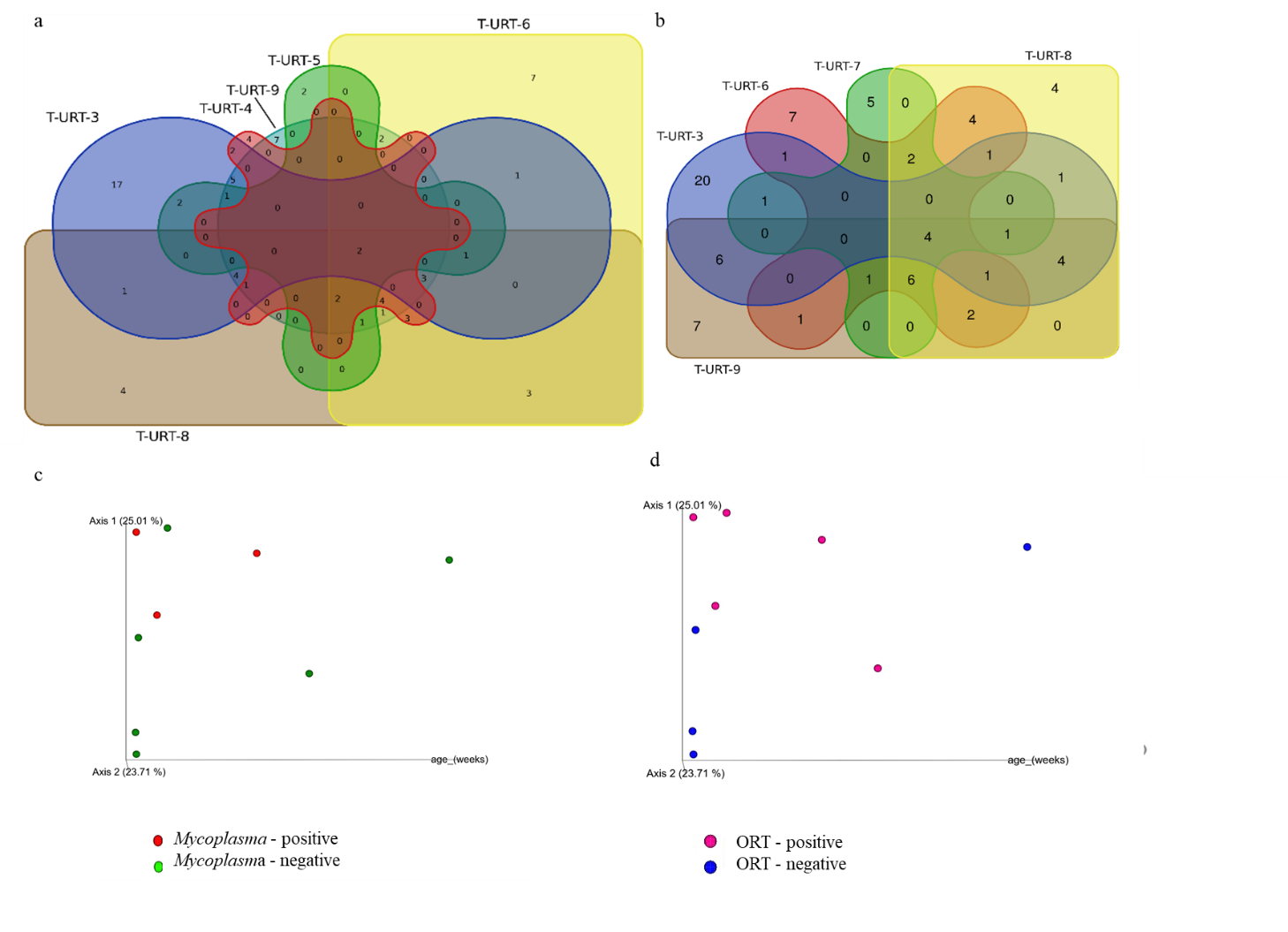


**a)** Venn diagram showing the number of OTUs at the genus level in flocks *Mycoplasma* positive (T-URT-6, T-URT-7, T-URT-9) and flocks *Mycoplasma* negative (T-URT-3, T-URT-4,T-URT-5). **b)** Venn diagram showing the number of OTUs at the genus level in flocks *Mycoplasma* positive (T-URT-6, T-URT-7, T-URT-9) and flocks *Ornithobacterium* positive (T-URT-3, T-URT-6,T-URT-7, T-URT-8). **c), d)** Clustering of URT of turkeys according to the occurrence of *Mycoplasma* (c) and ORT (d) in the microbiome. PCoA graph showing the significantly separate clustering by community composition of the bacterial communities in tracheal swabs from turkeys of different ages (AMOVA: P<0.001).
