# Supplementary material for: Bacterial communities of the upper respiratory tract of turkeys
Source: Sci Rep. 2021 Jan 28;11:2544. doi: 10.1038/s41598-021-81984-0 (PMC7843632; doi:10.1038/s41598-021-81984-0)
Supplement: Supplementary file 4 — Supplementary Information 4. [file 41598_2021_81984_MOESM4_ESM.html]

Javascript must be enabled to view this page.

magnitude
magnitudeUnassigned

200124\_2275.krona
200124\_2276.krona
200228\_2345.krona
200306\_2381.krona
200306\_2382.krona
200306\_2383.krona
200306\_2384.krona
200306\_2387.krona
200306\_2388.krona

6593962338142994147983113967157683188598129780150984

80213614090215

80213614090215

80213614090215

80213614090215

80213614090215

80213614090215

80213614090215

000000500

000000020

000800000

400000000

000020000

000040000

000400000

008000007

000060000

000400000

0013000008

000020000

000000200

0001700000

400000000

000300000

0000008300

004080000

004000000

004000000

004000000

004000000

004000000

004000000

004000000

000080000

000080000

000080000

000080000

000080000

000080000

000080000

6593162338142969147947113945157683188508129778150969

4001302373626827778664074910

4001302373626827778664074910

700000000

700000000

700000000

700000000

700000000

7070000000

7070000000

1000000000

1000000000

1000000000

6070000000

6070000000

6070000000

2651151231924527505601067150

1531140231924527505601067150

00231924506208578767050

00231924505674578700

00376001888197300

0082314203786381400

00010300000

00336000000

00784000000

00000534067050

00000534067050

11511060021297221100

11511060021191179100

000020000

0000000100

00000642200

00000112715700

11511060000000

000001064200

000001064200

38340000000

38340000000

38340000000

000000200

000000200

000000200

112110000000

112110000000

1100000000

1100000000

101110000000

500000000

96110000000

583600000170

583600000170

200000000

200000000

200000000

2350000000

2350000000

2350000000

2730000000

2730000000

2730000000

62800000170

6280000000

6280000000

0000000170

0000000170

010814172302736307590

000300500

000300500

000000500

000000500

000300000

000300000

0000000170

0000000170

0000000170

0000000170

040004812200

040004812200

000004812200

000004812200

040000000

040000000

000000500

000000500

000000500

000000500

000400000

000400000

000400000

000400000

010414171602254987420

010414171602254987420

0001600000

0001600000

00000249100

00000245500

0000003600

0000092700

0000092700

01041417001923807420

0012000000

067885000517400

000000700

005200018430500

0120000000

0000081000

000000700

000000020

0250000000

4187448151137297129174776266571912107342035138252

58575115000000

58575115000000

58575115000000

1292590000000

1292590000000

1292590000000

0011000000

0011000000

0011000000

12160000000

12160000000

12160000000

42460000000

42460000000

42460000000

4024304000000

1831154000000

1711090000000

1264000000

2193150000000

1352230000000

84920000000

01101300000

01101300000

0701300000

070000000

070000000

070000000

0001300000

0001300000

0001300000

040000000

040000000

040000000

040000000

204001755982102011309

204001755982102011309

260000000

200000000

200000000

200000000

060000000

060000000

060000000

500000000

500000000

500000000

500000000

19160000000

19160000000

19160000000

19160000000

792740000179800

792740000179800

792740000179800

0520000000

7316880000000

00000179800

4300000000

1800000000

6633990000000

5503340000000

5503340000000

0170000000

2891560000000

1451080000000

460000000

050000000

300000000

700000000

80320000000

22100000000

113650000000

113650000000

4800000000

59650000000

600000000

4584393189031109

35570000000

35570000000

35320000000

0250000000

8160600803

000000800

000000800

8160600003

8160000000

000600000

000000003

30540000000

30540000000

30540000000

28778000000

28770000000

0150000000

28620000000

008000000

008000000

500000000

500000000

500000000

79480000000

79480000000

79480000000

731380000000

731380000000

34800000000

39580000000

060000000

060000000

060000000

2081890000000

1931800000000

84790000000

040000000

87790000000

22180000000

1590000000

1590000000

1260000000

600000000

600000000

660000000

660000000

2631660000000

77260000000

59150000000

10110000000

800000000

1861400000000

94730000000

92670000000

1743230000300

070000000

070000000

5140000000

040000000

5100000000

942250000300

16460000000

000000300

9170000000

691190000000

0430000000

75770000000

75770000000

0530000000

0530000000

0530000000

28330000000

14160000000

14160000000

14170000000

14170000000

38380000000

1440000000

1440000000

17340000000

11220000000

6120000000

700000000

700000000

4584930000000

41230000000

41230000000

4074590000000

2533600000000

154990000000

10110000000

10110000000

18400000000

18400000000

18400000000

32150000000

32150000000

32150000000

500000000

500000000

500000000

238412690000000

238412690000000

16150000000

230000000

14233840000000

5140000000

080000000

080000000

090000000

850000000

3454030000000

070000000

28330000000

700000000

5120000000

86430000000

4900000000

0170000000

14220000000

020000000

0160000000

1771610000000

1000000000

2100000000

31260000000

070000000

82380000000

950000000

66310000000

070000000

070000000

070000000

1101180300000

1101180300000

070000000

0230000000

090000000

000300000

600000000

600000000

93700000000

590000000

1011520000000

1011520000000

090000000

700000000

5150000000

26340000000

63940000000

70920000000

70920000000

63920000000

700000000

540000000

540000000

540000000

4205370003006

46450000000

080000000

4600000000

0370000000

3744920003006

3013760003006

48720000000

25440000000

030000000

030000000

030000000

030000000

143351246401200400

1281120000400

1281120000400

1281120000400

43470000000

0100000000

0100000000

43370000000

43370000000

35390000000

35390000000

35390000000

706500000000

1400000000

1400000000

692500000000

692500000000

562200000000

562200000000

562200000000

2191840000000

31550000000

31550000000

2160290000000

38300000000

6400000000

5100000000

22600000000

1800000000

1418290000000

483870000000

483870000000

0210000000

11980000000

372080000000

0270000000

0170000000

0160000000

1121360000000

1121360000000

1121360000000

1100000000

1100000000

1100000000

1100000000

1100000000

1100000000

500000000

500000000

500000000

2955980000000

1372110000000

1372110000000

1583870000000

1583870000000

0200000000

0200000000

0200000000

3382560000000

3500000000

3500000000

3032560000000

89920000000

2141640000000

92940000000

92940000000

92870000000

070000000

927987120000000

927987120000000

622140000000

200000000

31900000000

4500000000

73215710000000

18200000000

16410000000

611190000000

3100000000

20600000000

192170000000

79290000000

29000000000

1956010000000

500000000

2346590000000

83623720000000

10100000000

173240000000

2094900000000

1900000000

8600000000

57860000000

3114700000000

060000000

15250000000

30900000000

30260000000

0170000000

4900000000

27591140000000

56614370000000

4800000000

1500000000

58610000000

2800000000

080000000

0220000000

1803060000000

641070000000

57800000000

934168001200000

55911050000000

55911050000000

37557501200000

0001200000

080000000

57490000000

43350000000

2000000000

2554220000000

0610000000

3800000000

3800000000

3800000000

13290000000

13290000000

13290000000

2088929830137274129140776266569912096042035138243

1955626462137197128327775286556512095239967138243

0003700000

0003700000

0003700000

0003700000

188292507320824645438996423257237071894

494536000000586

00000000586

00000000101

00000000485

4945360000000

4945360000000

183352453720824645438996423257237071308

18335245061784849179821698697595

000002145140

0000025636500

00000159174600

001362171223464322215

000530001330

0000000510

000900000

0000005400

0000015734200

005500292181932400

00000001290

183352450617600766163384990

0000010830200

001335797288244390

0000000450

0000017327200

000000200

0058001802571280

0000016720100

0032000000

0032000000

0310000000

0310000000

000366000102650

000366000102650

0075052130000

00003080000

0075049050000

0019101091982627136001303

00000004960

00000541974780

00000001890

0019101091928607424371303

0000250000

0000250000

0001400000

0001400000

0000000700

0000000700

0000000130

0000000130

0048618050000789

00170190000789

00170190000789

00149190000729

00210000060

00079400000

00079400000

00079400000

0031699200000

0031699200000

0031699200000

0061211182840000

0065300000

0002000000

0002000000

0063300000

0063300000

0003800000

0003800000

0002700000

0001100000

00002840000

00002840000

00002840000

000900000

000900000

000900000

00012098900000

00012098900000

0001000000

0002700000

0003800000

00011968100000

000123300000

0002900000

0002900000

000500000

0002400000

0131918739228000

0131918739228000

0131918739223000

000031400000

009204610000

00064570000

004310173000

0001300000

013002470000

0024000000

0032000000

000005000

000005000

000500000

000500000

000500000

000500000

7271376134432481167884555939769516260135560

7271376134432481167884555729769515093135523

5971234134432481166430555729769515093135523

00000124000

241352126363300449529762667071534135025

00105000000

002000000

00000002080

000001928232600

0032000000

001903000000

000002606294600

620001079733961152

000001793226500

601200002405312313560

0000014000

0016000000

000002447275300

0087614829931187813504020

00490000000

0000000630

0000170060

2907424414062818155125091179932

0000000660

000001418040

00000953120500

000159400000

00231650000254

1301420014540000

58730000000

0000630000

72690013910000

00000210116737

00000210116737

00000001200

00000210104737

133333687781398134820680

13332832776609860020680

000214019000

000214019000

0004200000

000153019000

0001900000

0004600000

0004600000

0004600000

13332832774009841020680

13332832774009841020680

1261490000000

114125370000015190

00120000450

000000080

000000050

0049400131402040

0000850000

00160000160

0000000390

000005000

000002202320

661460000000

0536014900000

0003600000

0003600000

0003600000

0004300000

0004300000

0004300000

01807000000

01807000000

000600000

0002700000

0003700000

0180000000

05180000000

05180000000

0400000000

0930000000

03850000000

0004074800

000400000

000400000

000400000

0000074800

0000074800

0000074800

2316712791986178613631759413603497669812706

3613835062633330

3433815000000

3433815000000

0015000000

0015000000

0015000000

343380000000

343380000000

500000000

1000000000

500000000

323380000000

11013000700

11013000700

500000000

500000000

500000000

6013000700

6013000700

600000000

0013000700

007062617330

007062617100

007000000

007000000

007000000

000062617100

000062617100

00006261700

0000000100

0000000230

0000000230

0000000230

0000000230

700000000

700000000

700000000

700000000

700000000

000000900

000000900

000000900

000000900

000000900

2280612753951178613631159385603167666512706

0065400000

006000000

006000000

006000000

006000000

0005400000

0005400000

0005400000

0005400000

497826205060

3170005060

1500000000

1500000000

1500000000

070000000

070000000

070000000

000000060

000000060

000000060

1100000000

1100000000

1100000000

500000000

500000000

500000000

000005000

000005000

000005000

1800000000

1800000000

1800000000

1800000000

00826200000

00826200000

00826200000

008000000

00026200000

00047884000000

00047884000000

00041382700000

0008923750000

0008923750000

00032458950000

00012520380000

00012928890000

000709680000

00001210000

00001210000

0000110000

0000670000

0000430000

0006590000

0006590000

0005290000

0001300000

00392900025544

00392900025544

00029000044

00029000044

00029000044

003900002550

003900002550

0039000000

0000000520

00000002030

10121391700717119937307489016

1012139581711961681748900

002744119371465748860

00270119371465748860

004000000

0000010000

000005935209244600

00230095131810

0000000250

0000112090100493630

0000000240

00000008330

00000124114300

0004400000

0004400000

000009500

000009500

000009500

1012112146258121140

1012112146255321140

000030000

00112000000

000002652900

0001400000

000000040

1000000000

0120000000

0000058000

000030000

0000010125400

00000121812800

0000028000

0000028000

0001694903249016

0001694903249016

0001694902149011

00000214900

000100000

000239200000

0001455000000

000100000

0005000011

000000005

000000005

0000011000

0000011000

30000235671359130

30000235671359130

300000000

300000000

300000000

00000235671359130

000001622955500

000001622955500

00000733880400

00000733880400

0000000130

0000000130

227441273475931278942382058227150112646

227441273475931278942382058227150112646

00530105201586726110

00530105201586726110

0000000450

0000000380

001504074492031370

0038058761093791410

000000511380

000000391120

00005700000

0035023131951320

0035023131951320

0035013791941190

00004670000

00004670000

00000010130

425001312581400

425001312581400

00001312581400

4250000000

0020001553220

0020001553220

0000000110

0020001553110

22662127224413183618977434184349359

22662127224413183618977434184349359

22362125303993183615931369274348786

0000071200

0000015119400

00000000191

3001924200288862790284

000000600

0000000098

00187041384229139584023287

00187041384229139584023287

0000490000

0000019355100

001370303246781802132818

0050011515695227189469

000036710000

40723087753646100

40723087753646100

0000180000

1600000000

2470000000

0023000000

000087573646100

000002000

000002000

000002000

000002000

000002000

000002000

4031834644024755438347111

22310000000

22310000000

22310000000

15230000000

15230000000

15230000000

780000000

780000000

780000000

180834644024755438347111

500000000

500000000

500000000

500000000

500000000

006000000

006000000

006000000

006000000

006000000

0031008611500

0031008611500

0031008611500

0031008611500

0031008611500

0060051602459632134420

0014000154000

0011100154000

0011100154000

00000129000

00111000000

0000025000

0029000000

0029000000

0029000000

0015831806000

0015831806000

0015831806000

007731806000

0081000000

000007000

000007000

000007000

000007000

001863017000

001863017000

001863017000

0002000000

001843017000

0028413502441232134420

0001100000

0001100000

0001100000

002844006034420

002844006034420

002204006034420

0064000000

000002440632100

000002440632100

000002440632100

0008400000

0008400000

0004600000

0003800000

1301971240732294

7000020000

7000020000

7000020000

700000000

0000020000

0000033200

0000033200

0000033200

0000033200

601971240200294

60197670200294

001876700000

001305400000

00571300000

0000000290

0000000290

60100020000

600000000

0010000000

0000020000

000000004

000000004

0005700000

0005700000

0005700000

000400007

000400007

000400007

000400007

000400007

090000000

090000000

090000000

090000000

070000000

070000000

070000000

020000000

020000000

020000000

00000183150

00000183150

00000183150

00000183150

00000183150

00000183150

0000016000

000002000

000000300

0000000150

448470000000

448470000000

448470000000

446470000000

446470000000

446470000000

423450000000

1620000000

700000000

200000000

200000000

200000000

200000000

070000000

070000000

070000000

070000000

070000000

070000000

070000000

001160005680

001160005680

001160005680

001160005680

001160005680

000000500

000000500

00116000000

00116000000

0000000680

0000000680

200000000

200000000

200000000

200000000

200000000

200000000

200000000
